# HIMA2: high-dimensional mediation analysis and its application in epigenome-wide DNA methylation data

**DOI:** 10.1186/s12859-022-04748-1

**Published:** 2022-07-25

**Authors:** Chamila Perera, Haixiang Zhang, Yinan Zheng, Lifang Hou, Annie Qu, Cheng Zheng, Ke Xie, Lei Liu

**Affiliations:** 1 Division of Biostatistics, Washington University in St. Louis, St. Louis, MO 63110 USA; 2 Center for Applied Mathematics, Tianjin University, Tianjin, 300072 China; 3 Department of Preventive Medicine, Northwestern University, Chicago, IL 60611 USA; 4 Department of Statistics, University of California, Irvine, CA 92697 USA; 5 Department of Biostatistics, University of Nebraska Medical Center, Omaha, NE 68198 USA

**Keywords:** Variable selection, Joint significant test, Epigenetics, Causality

## Abstract

Mediation analysis plays a major role in identifying significant mediators in the pathway between environmental exposures and health outcomes. With advanced data collection technology for large-scale studies, there has been growing research interest in developing methodology for high-dimensional mediation analysis. In this paper we present HIMA2, an extension of the HIMA method (Zhang in Bioinformatics 32:3150–3154, 2016). First, the proposed HIMA2 reduces the dimension of mediators to a manageable level based on the sure independence screening (SIS) method (Fan in J R Stat Soc Ser B 70:849–911, 2008). Second, a de-biased Lasso procedure is implemented for estimating regression parameters. Third, we use a multiple-testing procedure to accurately control the false discovery rate (FDR) when testing high-dimensional mediation hypotheses. We demonstrate its practical performance using Monte Carlo simulation studies and apply our method to identify DNA methylation markers which mediate the pathway from smoking to reduced lung function in the Coronary Artery Risk Development in Young Adults (CARDIA) Study.

**Supplementary Information:**

The online version contains supplementary material available at 10.1186/s12859-022-04748-1.

## Introduction

Mediation analysis explores the underlying mechanism by which an independent variable (e.g., exposure or treatment) influences the dependent variable (e.g., health outcome) through a mediator variable [1]. Mediation analysis has been playing a major role in many areas, e.g., social studies, economics, and health sciences [2]. More recently, with the advancement of large-scale data collection techniques, there has been substantial interest in developing methodology for high-dimensional mediation analysis in omics and imaging studies. An incomplete list of publications include [2–22]. For example, Derkach et al. [11] considered a latent variable model for high-dimensional mediation analysis. Huang et al. [12] presented a hypothesis test of the mediation effect in a causal mediation model with high-dimensional continuous mediators. Dai et al. [22] developed a multiple-testing procedure that accurately controls the false discovery rate (FDR) when testing high-dimensional mediation hypotheses.

Our motivating example comes from the DNA methylation (DNAm) research of the Coronary Artery Risk Development in Young Adults (CARDIA) Study [23]. In the DNAm process, methyl groups are added to DNA at binding sites referred to as cytosine-phosphate-guanine (CpG) islands, which inhibits the binding of transcription factors to DNA and results in changes (typically down regulation) to the expression of genes [24]. The platform Illumina MethylationEPIC Beadchip array is used to measure DNAm levels of roughly 850 K probes, which are ultra high-dimensional. Such high-dimensional DNAm markers may mediate pathways linking environmental exposures with health outcomes. Our objective is to explore the mediating role from high dimensional DNAm markers on the relationship between smoking and lung function in the CARDIA study.

In this paper, we propose an improved estimation and inference procedure for the high-dimensional mediation model, extending the work of Zhang et al. [3]. Our method includes three major steps: First, to tackle the ultra-high dimensionality of the DNAm markers, we screen out potentially a large number of mediators using a series of marginal mediation effect pathways (exposure $\to$ mediator $\to$ outcome). Second, we adopt the de-biased Lasso method [25] to estimate the high dimensional regression coefficients (mediator $\to$ outcome). Third, we employ a joint significance test with a mixture of null distributions to accurately control the FDR for large-scale multiple tests [22].

The remainder of this paper is structured as follows. In "Methodology" Section, we propose a three-step inference procedure for mediation effects in the high-dimensional regression model. In "Simulation studies" Section, we evaluate the performance of our method via numerical simulations. In "Application" Section, an application to the CARDIA study is provided. Finally, some discussion and concluding remarks are presented in "Conclusion and remarks" Section.

## Methodology

Denote the exposure as $X$, baseline covariates to be adjusted for as ${\varvec{Z}}={({Z}_{1},\dots ,{Z}_{q})}^{T}$, where the superscript $T$ denotes the transpose of a vector or a matrix. We adopt the following counterfactual framework for the vector of potential mediators ${\varvec{M}}(x)={({M}_{1}(x),\dots ,{M}_{p}(x))}^{T}$ under exposure level $x$, and counterfactual $Y(x,{\varvec{m}})$ under exposure level $x$ and mediators level ${\varvec{m}}$**,** to perform the mediation analysis [26]:

1’ $$ Y\left( {x,{\varvec{m}}} \right) = \gamma x + {\varvec{\beta}}^{{\varvec{T}}} {\varvec{m}} + {\varvec{\eta}}^{{\varvec{T}}} {\varvec{Z}} + \varepsilon $$

2’ $$ M_{j} \left( x \right) = \alpha_{j} x + {\varvec{\delta}}_{{\varvec{j}}}^{{\varvec{T}}} {\varvec{Z}} + e_{j} \;{\text{for}}\;j = 1, \ldots ,p $$

where $\gamma$ is the direct effect of exposure on the outcome; ${\varvec{\beta}}={({\beta }_{1},\dots ,{\beta }_{p})}^{T}$ is the regression parameter vector relating the mediators to the outcome; $\boldsymbol{\alpha }={({\alpha }_{1},\dots ,{\alpha }_{p})}^{T}$ is the parameter vector relating the exposure to mediators; ${\varvec{\eta}}$ and ${{\varvec{\delta}}}_{{\varvec{j}}}$ are vectors of regression coefficients for the covariates; and $\varepsilon$ and ${e}_{j}$ are error terms in Models (1’) and (2’), respectively. Note there are $p$ submodels in Model (2’), one for each mediator. We allow the correlation between the error terms, i.e., ${\varvec{e}}={({e}_{1},\dots ,{e}_{p})}^{T}\sim N(0,{\Sigma }_{e})$, where ${\Sigma }_{e}$ is a positive definite covariance matrix.

A few causal assumptions that are needed for the identification of natural direct effect (NDE) and natural indirect effects (NIE) are listed below [41–42]:

A1. Stable unit treatment value assumption (SUTVA) for both the mediators and the outcome. This assumption means that there is no multiple versions of exposures and there is no interference between individuals, which implies that ${\varvec{M}}(x)$ and $Y(x,{\varvec{m}})$ are well defined.

A2. Consistency for the mediators and the outcome. That is, there are no measurement errors in the mediators and thus the observed variables satisfy ${\varvec{M}}={\varvec{M}}(X)$ and $Y=Y(X,{\varvec{M}})$.

A3. Sequential ignorability: This assumption contains 4 parts:

(A3.1) $X\perp Y(x,{\varvec{m}})|{\varvec{Z}}$**,** i.e., no unmeasured confounding between exposure and the potential outcome;

(A3.2) ${\varvec{M}}\perp Y(x,{\varvec{m}})|X,{\varvec{Z}}$**,** i.e., no unmeasured confounding between mediators and the potential outcome;

(A3.3) $X\perp {\varvec{M}}(x)|{\varvec{Z}}$**,** i.e., no unmeasured confounding between exposure and the potential mediators;

(A3.4) ${\varvec{M}}\left( {x^{\prime}} \right) \bot Y\left( {x,{\varvec{m}}} \right)|{\varvec{Z}}$**,** i.e., no exposure-induced confounding between mediators and the potential outcome. In other words, the potential mediators under any intervention level $\varvec{m}$ are independent of potential outcomes under any intervention *x* and mediator level $x^{\prime}$ given covariate $\varvec{Z}$.

A4. No direct causal relationship between mediators. We do not allow one mediator to be the cause of another, but we do allow them to have shared common causes.

Under A1-A3, we have direct effect $NDE=E\left[Y\left(1,{\varvec{M}}(0)\right)-Y\left(0,{\varvec{M}}(0)\right)\right]=\gamma$, indirect effect $NIE=E\left[Y\left(1,{\varvec{M}}(1)\right)-Y\left(1,{\varvec{M}}(0)\right)\right]=\sum_{j=1}^{p}{\alpha }_{j}{\beta }_{j}$. Under the additional assumption A4, we can decompose the indirect effect into sum of indirect effects through each mediator ${M}_{j}$, ${NIE}_{j}={\alpha }_{j}{\beta }_{j}$. Also we obtain the structural equation model for the observed outcome as in previous literature [3] to assess the mediation effects of high-dimensional mediators:

1 $$ Y = \gamma X + {\varvec{\beta}}^{\varvec{T}} {\varvec{M}} + {\varvec{\eta}}^{{\varvec{T}}} {\varvec{Z}} + \varepsilon , $$

2 $$ M_{j} = {\alpha_{j}} X + {\varvec{\delta}}_{\varvec{j}}^{\varvec{T}} {\varvec{Z}} + {e_{j}} \, \,{\text{for}} \, j \,= \,1, \, \ldots, \, p, $$

**Fig. 1:**
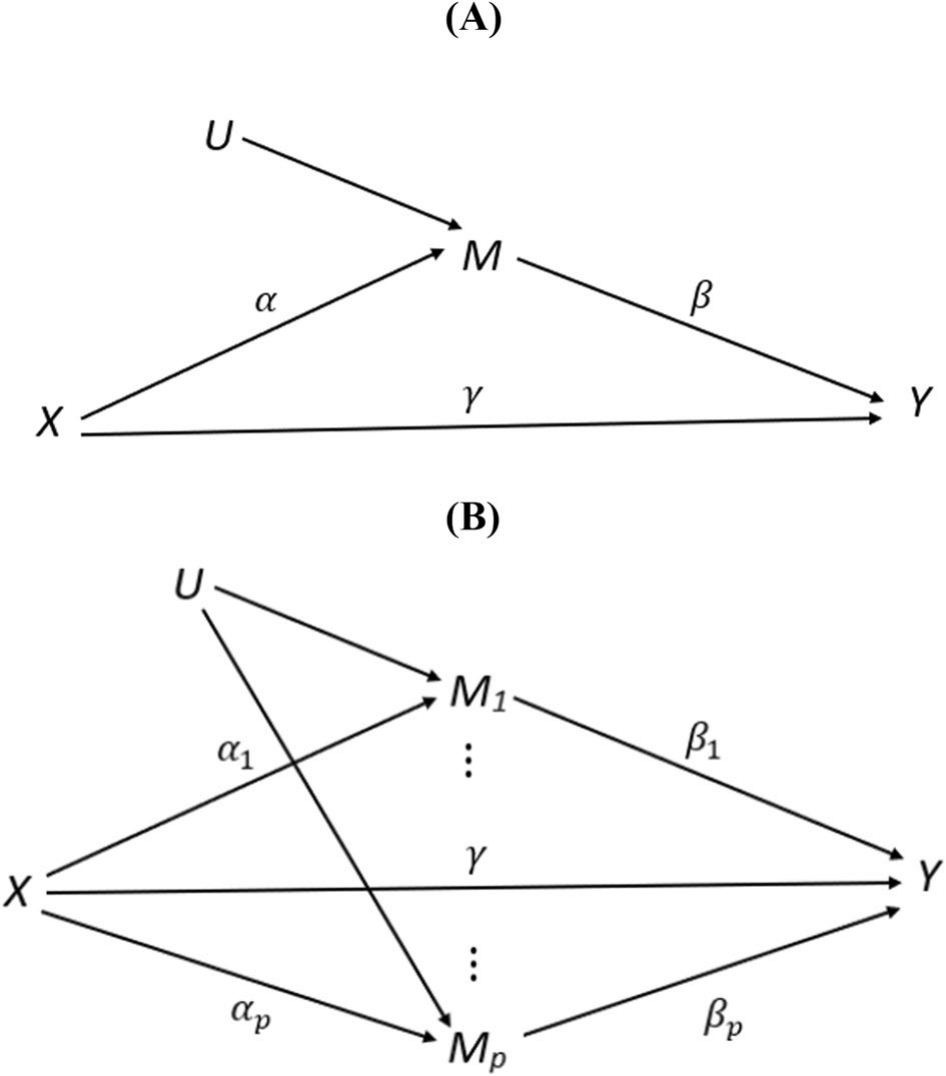
Mediation analysis of **A** a single mediator; **B** high dimensional mediators, plotted similarly to [3]. An arrow from $X$ to $U$ is possible though omitted to avoid the complexity in interpreting $\alpha$ as the total effect

Our goal is to estimate and test the mediation effects ${\alpha }_{j}{\beta }_{j}$ jointly for $j=1,\dots ,p.$ An illustration of mediation analyses with single mediator and high dimensional mediators is given in Fig. 1.

As shown in Fig. 1, we do allow these mediators to share common unmeasured causes. These assumptions are in line with the underlying biologic procedures. Smoking could induce biochemical alterations to the DNAs, which lead to methylation changes. Such change in a certain CpG site is unlikely to *directly* cause the methylation alternation of other CpG sites. Rather, such dependency is most likely to be indirect, for example, by regulating gene expressions in related pathways that in turn modify other CpGs, or several CpGs are modified by common unmeasured causes (e.g., inflammatory response).

Details of our proposed approach are given below.

*Step 1*: (*Screening of Mediators*). For $j=1,\dots ,p$, we consider a series of marginal models:

3 $$ Y = \gamma X + \beta_{j} M_{j} + {\varvec{\eta}}^{{\varvec{T}}} {\varvec{Z}} + \varepsilon $$

4 $$ M_{j} = \alpha_{j} X + {\varvec{\delta}}_{{\varvec{j}}}^{{\varvec{T}}} {\varvec{Z}} + e_{j} $$

Along the lines of the sure independence screening (SIS) method [27], we select a subset $\mathcal{D}=\{j : {M}_{j}$ is among the top $d=\left[2n/\mathrm{log}(n)\right]$ largest effect$\left|{\widehat{\alpha }}_{j}{\widehat{\beta }}_{j}\right|$, for$j=1,\dots ,p\}$, where ${\widehat{\alpha }}_{j}$ and ${\widehat{\beta }}_{j}$ are ordinary least square (OLS) estimators based on marginal models (3) and (4), respectively.

All ${M}_{j}$'s are scaled with mean zero and unit variance before performing this screening procedure. The key advantage of Step 1 is that the product term ${\widehat{\alpha }}_{j}{\widehat{\beta }}_{j}$ could roughly describe the mediated effect of the $j$ th mediator. Therefore, the selected subset $\mathcal{D}$ contains true mediators with a large probability.

*Step 2*: (*De-biased Lasso*). We consider the following submodel based on the selected set $\mathcal{D}$,

5 $$ Y = \gamma X + {\varvec{\beta}}_{{\mathbf{\mathcal{D}}}}^{{\varvec{T}}} {\varvec{M}}_{{\mathbf{\mathcal{D}}}} + {\varvec{\eta}}^{{\varvec{T}}} {\varvec{Z}} + \varepsilon $$

where ${{\varvec{\beta}}}_{\mathcal{D}}$ and ${{\varvec{M}}}_{\mathcal{D}}$ denote sub-vectors of ${\varvec{\beta}}$ and ${\varvec{M}}$ with index belonging to $\mathcal{D}$ respectively, and ${{\varvec{\beta}}}_{\mathcal{D}}$ is estimated using the de-biased Lasso method, with estimator ${\widehat{\beta }}_{j}$ and its standard error ${\widehat{\sigma }}_{{\beta }_{j}}$ obtained in [25]. The corresponding p-values are given as:

6 $$  P_{{\beta_{j} }} = 2\left\{ {1 - {\Phi }\left( {\left| {\hat{\beta }_{j} } \right|/{ }\hat{\sigma }_{{\beta_{j} }} } \right)} \right\},\;{\hbox{for}}\;j \mathcal{D} $$

where $\Phi \left(\cdot \right)$ is the cumulative distribution function of $N\left(\mathrm{0,1}\right).$ De-biased Lasso in Step 2 is necessary as the ordinary least square will yield inefficient estimates (with reduced power), because the dimension of survived mediators after Step 1 is still relatively large.

*Step 3:* (*Joint Significance Test*). We consider the multiple testing problem for $j \epsilon \mathcal{D}$ as follows:

$$ H_{0j} : \alpha_{j} = 0\;{\text{or}}\;\beta_{j} = 0, $$

with corresponding p-value

7 $$ P_{j} = {\text{max}}\left( {P_{{\alpha_{j} }} ,P_{{\beta_{j} }} } \right) $$

where ${P}_{{\beta }_{j}}$ is given in (6), ${P}_{{\alpha }_{j}}=2\left\{1-\Phi \left(\left|{\widehat{\alpha }}_{j}\right|/ {\widehat{\sigma }}_{{\alpha }_{j}}\right)\right\}$, ${\widehat{\alpha }}_{j}$ and ${\widehat{\sigma }}_{{\alpha }_{j}}$ are OLS estimators. Zhang et al. [3] considered the joint significant test (termed “JS-uniform”), which assumes that ${P}_{j}$ follows a uniform distribution. However, although ${P}_{{\alpha }_{j}}$ and ${P}_{{\beta }_{j}}$ are each uniformly distributed, their maximum is not. As a result, the significance rule using the uniform null distribution for ${P}_{j}$ results in a valid but overly conservative test [28]. In this paper, we will adopt the “JS-mixture" approach to accurately control the FDR [22] (Sect. 2.3).

The multiple testing problem (7) is equivalent to the union of the following three disjoint component null hypotheses,

$$ H_{00,j} : \alpha_{j} = 0\;{\text{or}}\;\beta_{j} = 0, $$

$$ H_{01,j} : \alpha_{j} = 0\;{\text{or}}\;\beta_{j} \ne 0, $$

$$ H_{10,j} : \alpha_{j} \ne 0\;{\text{or}}\;\beta_{j} = 0. $$

That is, ${P}_{j}$ is a 3-component mixture distribution instead of the uniform distribution. Dai et al. [22] proposed the following estimated FDR for testing mediation:

8 $$ \widehat{FDR}\left( t \right) = \frac{{\hat{\pi }_{01} t + \hat{\pi }_{10} t + \hat{\pi }_{00} t^{2} }}{{{\text{max}}\left\{ {1,R\left( t \right)} \right\}/d}} $$

where ${\widehat{\pi }}_{01}, {\widehat{\pi }}_{10}$ and ${\widehat{\pi }}_{00}$ are the estimates of proportions ${H}_{01,j}, {H}_{10,j}$ and ${H}_{00,j}$, respectively, and $R\left(t\right)={V}_{00}\left(t\right)+{V}_{01}\left(t\right)+$ ${V}_{10}\left(t\right)+$ ${V}_{11}\left(t\right)$, where ${V}_{00}\left(t\right)=\#\left\{{P}_{j}\le t|{H}_{00}\right\}$, ${V}_{01}\left(t\right)=\#\left\{{P}_{j}\le t|{H}_{01}\right\}$, ${V}_{10}\left(t\right)=\#\left\{{P}_{j}\le t|{H}_{10}\right\}$, ${V}_{11}\left(t\right)=\#\left\{{P}_{j}\le t|{H}_{11}\right\}$ for $t\in \left[\mathrm{0,1}\right].$

We define the significant threshold for ${P}_{j}$ as ${\widehat{t}}_{b}=\mathrm{sup}\left\{t: \widehat{FDR}(t)\le b\right\},$ to control the FDR at level $b$. Then $\widehat{S}=\left\{j:{P}_{j}\le {\widehat{t}}_{b}, j\in \mathcal{D}\right\}$ gives the estimated index set of significant mediators.

We can obtain ${\widehat{\pi }}_{01}, {\widehat{\pi }}_{10}$, ${\widehat{\pi }}_{00}$ and ${\widehat{t}}_{b}$ using the R package HDMT [22].

Compared to the estimation and inference method in [3] (termed “HIMA”), our new method (termed “HIMA2”) has the following three advantages. First, HIMA only considers $\beta$ (mediator $\to$ outcome) for screening in Step 1, while HIMA2 considers the indirect effect of $\alpha \beta$. Therefore, the mediation-based screening method in HIMA2 addresses the indirect effect more accurately than HIMA. Second, HIMA uses the minimax concave penalty (MCP; [29]) technique to estimate the effect $\beta$, which can only provide p-values for selected mediators in Step 2. That is, ${P}_{{\beta }_{j}}$ is set to 1 for those not selected, which results in poor estimate of ${P}_{j}$ in Eq. (7). In contrast, de-biased Lasso in HIMA2 yields p-values for all ${\beta }_{j}$’s in $\mathcal{D}$, which gives more appropriate estimate of ${P}_{{\beta }_{j}}$. Third, HIMA adopts a naive joint significance rule assuming a uniform null distribution for the maximum p-value calculation in Step 3, which may result in a valid but overly conservative test with lower power.

## Simulation studies

In this section we assess our proposed method using simulation studies. For Model (1), we generate the exposure $X$ from $N(0, 2)$; covariates $Z={\left({Z}_{1}, {Z}_{2}\right)}^{T}$, where ${Z}_{1}$ and ${Z}_{2}$ are independently generated from $N(0, 2)$. We set $\gamma =0.5$, $\delta ={\left(0.\mathrm{3,0.3}\right)}^{T}$ and $\eta ={\left(0.5, 0.5\right)}^{T}$; ${\beta }_{1}=0.20,$ ${\beta }_{2}=0.25,$ ${\beta }_{3}=0.15,$ ${\beta }_{4}=0.30,$ ${\beta }_{5}=0.35,$ ${\beta }_{6}=0.10,$ and ${\beta }_{j}=0$ for all other $j$’s; ${\alpha }_{1}=0.20,$ ${\alpha }_{2}=0.25,$ ${\alpha }_{3}=0.15,$ ${\alpha }_{4}=0.30,$ ${\alpha }_{5}=0.35,$ ${\alpha }_{7}=0.10,$ and ${\alpha }_{j}=0$ for all other $j$’s. Therefore, we have: (i) ${\alpha }_{j}{\beta }_{j}\ne 0$ for $j=1,\dots ,5$; (ii) ${\alpha }_{j}=0$ but ${\beta }_{j}\ne 0$ for $j=6$; (iii) ${\alpha }_{j}\ne 0$ but ${\beta }_{j}=0$ for $j=7$; and (iv) ${\alpha }_{j}=0$ and ${\beta }_{j}=0$ for $j>7$. The error terms $e={\left({e}_{1}, \dots ,{e}_{p}\right)}^{T}$ are generated from $N(0,{\Sigma }_{e})$, where ${\Sigma }_{e}={\left({\rho }^{|j-{j}^{^{\prime}}|}\right)}_{j,{j}^{^{\prime}}}$ and $\varepsilon$ is generated from $N(0, 1)$. All the simulations are based on 500 replications with 16 factorial settings: $p=1000, 5000$, $n=300,$ 600, and $\rho =0, 0.25, 0.5, 0.75$.

We compare the performance of HIMA2 with HIMA in Table 1, which provides the estimated biases (Bias) given by the sample mean of the estimates minus the true value, and the mean-square error (MSE) of the estimates. Table 1 shows that both HIMA2 and HIMA are unbiased, however, HIMA2 has smaller MSEs than HIMA for significant mediators. MSEs for both HIMA and HIMA2 decrease as the sample size increases. Of note, the results for $j>8$ (${\alpha }_{j}=0$ and ${\beta }_{j}=0$) are close to those of $j=8$ and thus omitted.

**Table 1:** Bias (MSE) for mediation effect estimates

		$$\rho =0$$					
		$p=1000$			$p=5000$		
		HIMA2	HIMA	HDMA	HIMA2	HIMA	HDMA
$n=300$	${\alpha }_{1}{\beta }_{1}$	7.21E−04 (1.72E−04)	−7.07E−03 (4.23E−04)	−8.95E−03 (2.80E−04)	−8.90E−03 (1.97E−04)	−2.23E−02 (8.19E−04)	−2.25E−02 (7.14E−04)
	${\alpha }_{2}{\beta }_{2}$	−2.44E−04 (2.80E−04)	−7.27E−03 (5.10E−04)	−1.30E−02 (4.28E−04)	−1.34E−02 (4.12E−04)	−2.71E−02 (1.22E−03)	−2.90E−02 (1.09E−03)
	${\alpha }_{3}{\beta }_{3}$	9.45E−04 (1.00E−04)	−8.32E−03 (2.84E−04)	−7.54E−03 (1.91E−04)	−4.17E−03 (1.17E−04)	−1.43E−02 (3.63E−04)	−1.40E−02 (3.00E−04)
	${\alpha }_{4}{\beta }_{4}$	−2.22E−03 (4.86E−04)	−1.16E−02 (7.75E−04)	−1.95E−02 (8.11E−04)	−1.87E−02 (6.97E−04)	−3.27E−02 (1.52E−03)	−3.89E−02 (1.77E−03)
	${\alpha }_{5}{\beta }_{5}$	−4.82E−03 (5.89E−04)	−1.63E−02 (1.03E−03)	−2.66E−02 (1.23E−03)	−2.51E−02 (1.07E−03)	−4.48E−02 (2.66E−03)	−5.21E−02 (3.04E−03)
	${\alpha }_{6}{\beta }_{6}$	6.06E−05 (1.05E−05)	−3.76E−05 (8.92E−06)	4.81E−05 (7.21E−06)	5.10E−05 (4.32E−06)	3.56E−05 (1.62E−06)	2.88E−05 (1.57E−06)
	${\alpha }_{7}{\beta }_{7}$	2.41E−03 (3.71E−05)	3.21E−04 (5.91E−06)	3.75E−04 (8.43E−06)	1.13E−03 (1.81E−05)	5.43E−05 (1.85E−06)	6.02E−05 (1.60E−06)
	${\alpha }_{8}{\beta }_{8}$	1.11E−04 (1.08E−06)	1.17E−05 (1.09E−07)	3.62E−05 (2.72E−07)	4.58E−05 (4.02E−07)	−1.03E−05 (4.31E−08)	−5.98E−06 (3.04E−08)
$n=600$	${\alpha }_{1}{\beta }_{1}$	1.46E−03 (1.01E−04)	2.88E−03 (1.64E−04)	−4.34E−03 (1.06E−04)	−6.78E−03 (1.18E−04)	−9.43E−03 (2.22E−04)	−1.49E−02 (2.89E−04)
	${\alpha }_{2}{\beta }_{2}$	1.00E−03 (1.59E−04)	3.47E−03 (2.45E−04)	−7.38E−03 (2.00E−04)	−1.01E−02 (2.23E−04)	−1.32E−02 (3.40E−04)	−2.26E−02 (6.03E−04)
	${\alpha }_{3}{\beta }_{3}$	8.87E−04 (4.72E−05)	−2.49E−04 (1.38E−04)	−3.13E−03 (6.45E−05)	−3.22E−03 (5.42E−05)	−7.51E−03 (1.67E−04)	−9.34E−03 (1.40E−04)
	${\alpha }_{4}{\beta }_{4}$	7.51E−04 (1.97E−04)	4.32E−03 (2.97E−04)	−1.03E−02 (2.94E−04)	−1.44E−02 (4.12E−04)	−1.90E−02 (5.96E−04)	−3.22E−02 (1.18E−03)
	${\alpha }_{5}{\beta }_{5}$	−1.03E−03 (2.80E−04)	2.39E−03 (4.03E−04)	−1.59E−02 (5.36E−04)	−2.12E−02 (7.05E−04)	−2.89E−02 (1.13E−03)	−4.57E−02 (2.27E−03)
	${\alpha }_{6}{\beta }_{6}$	1.15E−05 (4.16E−06)	−9.97E−05 (4.28E−06)	−3.24E−05 (3.21E−06)	1.59E−04 (2.27E−06)	1.43E−04 (2.06E−06)	1.37E−04 (1.45E−06)
	${\alpha }_{7}{\beta }_{7}$	2.20E−03 (2.09E−05)	1.29E−04 (1.32E−06)	2.99E−04 (7.03E−06)	6.68E−04 (1.07E−05)	4.24E−05 (1.28E−06)	1.01E−04 (1.29E−06)
	${\alpha }_{8}{\beta }_{8}$	−4.17E−06 (4.54E−07)	3.83E−06 (7.98E−08)	−4.66E−06 (2.06E−07)	−6.36E−06 (1.62E−07)	−4.77E−06 (9.88E−09)	5.34E−06 (4.52E−08)

		$$\rho =0.25$$					
		$p=1000$			$p=5000$		
		HIMA2	HIMA	HDMA	HIMA2	HIMA	HDMA
$n=300$	${\alpha }_{1}{\beta }_{1}$	4.46E−04 (1.82E−04)	−7.65E−03 (4.95E−04)	−7.79E−03 (2.49E−04)	−6.92E−03 (1.95E−04)	−1.85E−02 (6.84E−04)	−1.84E−02 (5.13E−04)
	${\alpha }_{2}{\beta }_{2}$	3.22E−04 (3.08E−04)	−6.70E−03 (5.90E−04)	−1.07E−02 (3.88E−04)	−1.07E−02 (3.65E−04)	−2.17E−02 (9.31E−04)	−2.58E−02 (8.66E−04)
	${\alpha }_{3}{\beta }_{3}$	5.90E−04 (1.09E−04)	−7.05E−03 (2.83E−04)	−4.87E−03 (1.40E−04)	−3.934E−03 (1.01E−04)	−1.33E−02 (3.34E−04)	−1.22E−02 (2.38E−04)
	${\alpha }_{4}{\beta }_{4}$	−2.44E−03 (4.26E−04)	−1.04E−02 (7.13E−04)	−1.74E−02 (7.17E−04)	−1.81E−02 (6.81E−04)	−3.33E−02 (1.69E−03)	−3.79E−02 (1.74E−03)
	${\alpha }_{5}{\beta }_{5}$	−3.57E−03 (5.93E−04)	−1.53E−02 (1.02E−03)	−2.34E−02 (1.15E−03)	−2.25E−02 (9.91E−04)	−4.21E−02 (2.39E−03)	−5.08E−02 (2.94E−03)
	${\alpha }_{6}{\beta }_{6}$	1.77E−04 (9.38E−06)	1.56E−04 (7.85E−06)	2.24E−04 (7.31E−06)	−4.44E−05 (4.70E−06)	2.45E−05 (2.84E−06)	−1.12E−05 (2.35E−06)
	${\alpha }_{7}{\beta }_{7}$	3.63E−03 (4.74E−05)	4.27E−04 (1.09E−05)	7.49E−04 (1.07E−05)	1.89E−03 (2.29E−05)	1.84E−04 (3.29E−06)	2.20E−04 (3.13E−06)
	${\alpha }_{8}{\beta }_{8}$	7.33E−05 (9.05E−07)	−2.12E−05 (1.81E−07)	−9.48E−06 (2.56E−07)	3.63E−05 (4.97E−07)	−1.24E−05 (3.90E−08)	6.43E−07 (2.70E−08)
$n=600$	${\alpha }_{1}{\beta }_{1}$	8.96E−04 (9.43E−05)	2.08E−03 (1.75E−04)	−3.94E−03 (1.07E−04)	−6.44E−03 (1.24E−04)	−8.81E−03 (1.98E−04)	−1.44E−02 (2.69E−04)
	${\alpha }_{2}{\beta }_{2}$	3.74E−04 (1.50E−04)	2.77E−03 (2.21E−04)	−6.33E−03 (1.95E−04)	−9.28E−03 (2.15E−04)	−1.32E−02 (3.50E−04)	−2.20E−02 (5.88E−04)
	${\alpha }_{3}{\beta }_{3}$	1.41E−03 (5.39E−05)	1.33E−04 (1.31E−04)	−1.94E−03 (5.17E−05)	−3.45E−03 (5.67E−05)	−6.69E−03 (1.41E−04)	−8.19E−03 (1.06E−04)
	${\alpha }_{4}{\beta }_{4}$	3.66E−04 (2.13E−04)	3.32E−03 (2.95E−04)	−8.58E−03 (2.91E−04)	−1.35E−02 (3.75E−04)	−1.93E−02 (5.98E−04)	−3.14E−02 (1.13E−03)
	${\alpha }_{5}{\beta }_{5}$	4.79E−04 (3.29E−04)	3.09E−03 (4.39E−04)	−1.15E−02 (4.74E−04)	−1.95E−02 (6.63E−04)	−2.81E−02 (1.11E−03)	−4.37E−02 (2.12E−03)
	${\alpha }_{6}{\beta }_{6}$	−2.74E−05 (3.95E−06)	−1.16E−04 (4.39E−06)	−2.32E−05 (3.27E−06)	−1.33E−04 (2.97E−06)	−1.96E−04 (2.85E−06)	−6.82E−05 (1.90E−06)
	${\alpha }_{7}{\beta }_{7}$	3.17E−03 (2.65E−05)	3.37E−04 (4.72E−06)	1.13E−03 (8.65E−06)	2.24E−03 (1.90E−05)	1.23E−04 (1.92E−06)	2.37E−04 (2.55E−06)
	${\alpha }_{8}{\beta }_{8}$	1.62E−05 (3.35E−07)	−5.68E−07 (4.13E−08)	−6.73E−07 (2.26E−07)	2.72E−05 (1.54E−07)	1.69E−05 (6.54E−08)	1.30E−05 (2.72E−08)

		$$\rho =0.50$$					
		$p=1000$			$p=5000$		
		HIMA2	HIMA	HDMA	HIMA2	HIMA	HDMA
$n=300$	${\alpha }_{1}{\beta }_{1}$	3.76E−03 (2.34E−04)	−3.46E−03 (4.90E−04)	−1.78E−03 (2.13E−04)	−5.14E−03 (1.98E−04)	−1.63E−02 (6.36E−04)	−1.46E−02 (3.74E−04)
	${\alpha }_{2}{\beta }_{2}$	2.82E−03 (3.66E−04)	−5.79E−03 (8.16E−04)	−4.11E−03 (3.79E−04)	−7.97E−03 (3.72E−04)	−2.14E−02 (1.13E−03)	−2.18E−02 (7.77E−04)
	${\alpha }_{3}{\beta }_{3}$	2.25E−03 (1.42E−04)	−6.91E−03 (3.36E−04)	−9.13E−04 (1.19E−04)	−3.27E−03 (1.19E−04)	−1.32E−02 (3.33E−04)	−8.64E−03 (1.59E−04)
	${\alpha }_{4}{\beta }_{4}$	1.03E−03 (5.02E−04)	−8.62E−03 (9.89E−04)	−7.04E−03 (5.47E−04)	−1.15E−02 (5.83E−04)	−2.90E−02 (1.65E−03)	−2.97E−02 (1.26E−03)
	${\alpha }_{5}{\beta }_{5}$	6.66E−03 (7.53E−04)	−5.38E−03 (1.01E−03)	−7.22E−03 (7.94E−04)	−1.27E−02 (7.87E−04)	−3.52E−02 (2.06E−03)	−4.02E−02 (2.24E−03)
	${\alpha }_{6}{\beta }_{6}$	−3.47E−05 (8.66E−06)	−2.58E−04 (7.30E−06)	9.43E−05 (7.46E−06)	6.55E−05 (6.88E−06)	6.48E−05 (6.34E−06)	7.46E−05 (5.69E−06)
	${\alpha }_{7}{\beta }_{7}$	4.69E−03 (5.74E−05)	9.18E−04 (1.63E−05)	2.09E−03 (2.36E−05)	3.81E−03 (4.57E−05)	5.83E−04 (9.04E−06)	1.06E−03 (1.21E−05)
	${\alpha }_{8}{\beta }_{8}$	1.82E−05 (1.91E−06)	−3.56E−05 (5.92E−07)	−1.17E−05 (7.80E−07)	−1.28E−05 (4.19E−07)	−2.13E−05 (1.67E−07)	1.08E−05 (1.89E−07)
$n=600$	${\alpha }_{1}{\beta }_{1}$	2.44E−03 (1.08E−04)	2.14E−03 (1.74E−04)	4.14E−04 (1.01E−04)	−3.50E−03 (1.01E−04)	−6.87E−03 (1.99E−04)	−1.07E−02 (1.95E−04)
	${\alpha }_{2}{\beta }_{2}$	3.41E−03 (2.02E−04)	4.48E−03 (2.75E−04)	7.19E−04 (1.71E−04)	−5.51E−03 (2.26E−04)	−9.64E−03 (3.32E−04)	−1.68E−02 (4.46E−04)
	${\alpha }_{3}{\beta }_{3}$	1.76E−03 (7.22E−05)	−3.66E−03 (1.91E−04)	6.19E−04 (6.90E−05)	−1.24E−03 (6.31E−05)	−6.00E−03 (1.74E−04)	−5.34E−03 (7.98E−05)
	${\alpha }_{4}{\beta }_{4}$	4.34E−03 (2.65E−04)	4.77E−03 (3.36E−04)	2.98E−04 (2.42E−04)	−9.92E−03 (3.48E−04)	−1.59E−02 (5.71E−04)	−2.48E−02 (8.05E−04)
	${\alpha }_{5}{\beta }_{5}$	8.04E−03 (4.34E−04)	9.29E−03 (5.06E−04)	1.81E−03 (3.96E−04)	−9.00E−03 (4.37E−04)	−1.82E−02 (7.82E−04)	−3.04E−02 (1.23E−03)
	${\alpha }_{6}{\beta }_{6}$	1.10E−04 (4.60E−06)	5.09E−05 (3.85E−06)	9.57E−05 (4.21E−06)	−1.66E−04 (3.28E−06)	−1.12E−04 (2.79E−06)	−7.62E−05 (2.46E−06)
	${\alpha }_{7}{\beta }_{7}$	2.91E−03 (3.12E−05)	8.64E−04 (1.22E−05)	2.17E−03 (2.10E−05)	3.23E−03 (2.66E−05)	6.05E−04 (6.67E−06)	1.23E−03 (9.06E−06)
	${\alpha }_{8}{\beta }_{8}$	6.18E−06 (4.08E−07)	−1.44E−06 (1.23E−07)	−2.07E−06 (4.32E−07)	2.24E−05 (2.45E−07)	1.13E−05 (5.31E−08)	5.00E−06 (1.28E−07)

		$$\rho =0.75$$					
		$p=1000$			$p=5000$		
		HIMA2	HIMA	HDMA	HIMA2	HIMA	HDMA
$n=300$	${\alpha }_{1}{\beta }_{1}$	7.39E−03 (3.47E−04)	−2.75E−03 (7.68E−04)	4.38E−03 (3.12E−04)	2.65E−03 (2.98E−04)	−8.93E−03 (7.81E−04)	−4.19E−03 (2.82E−04)
	${\alpha }_{2}{\beta }_{2}$	8.32E−03 (7.65E−04)	−1.43E−02 (2.29E−03)	5.21E−03 (6.63E−04)	2.17E−03 (5.13E−04)	−2.20E−02 (2.15E−03)	−8.00E−03 (6.11E−04)
	${\alpha }_{3}{\beta }_{3}$	4.03E−03 (2.70E−04)	−1.05E−02 (5.41E−04)	2.83E−03 (2.53E−04)	8.24E−05 (2.15E−04)	−1.32E−02 (5.05E−04)	−3.20E−03 (2.06E−04)
	${\alpha }_{4}{\beta }_{4}$	1.07E−02 (1.07E−03)	−6.93E−03 (2.81E−03)	5.83E−03 (9.77E−04)	1.03E−03 (7.32E−04)	−2.05E−02 (2.85E−03)	−1.21E−02 (9.51E−04)
	${\alpha }_{5}{\beta }_{5}$	1.74E−02 (1.50E−03)	−1.48E−02 (4.46E−03)	1.08E−02 (1.33E−03)	7.51E−03 (1.09E−03)	−3.15E−02 (4.61E−03)	−1.28E−02 (1.26E−03)
	${\alpha }_{6}{\beta }_{6}$	2.06E−05 (8.13E−06)	−1.05E−04 (6.97E−06)	4.44E−05 (8.01E−06)	9.89E−06 (7.72E−06)	−1.27E−04 (6.67E−06)	6.18E−05 (7.25E−06)
	${\alpha }_{7}{\beta }_{7}$	6.19E−03 (1.20E−04)	1.66E−03 (4.07E−05)	4.97E−03 (1.08E−04)	4.63E−03 (8.72E−05)	9.38E−04 (1.99E−05)	2.55E−03 (7.09E−05)
	${\alpha }_{8}{\beta }_{8}$	−1.15E−04 (5.76E−06)	−8.81E−06 (8.67E−07)	−1.04E−04 (5.11E−06)	−1.78E−05 (2.51E−06)	−5.95E−06 (1.02E−06)	−6.78E−05 (2.77E−06)
$n=600$	${\alpha }_{1}{\beta }_{1}$	4.81E−03 (1.64E−04)	8.99E−04 (4.05E−04)	3.88E−03 (1.57E−04)	1.75E−03 (1.52E−04)	−2.42E−03 (3.86E−04)	−3.55E−03 (1.46E−04)
	${\alpha }_{2}{\beta }_{2}$	8.29E−03 (4.00E−04)	−2.07E−03 (1.30E−03)	6.75E−03 (3.79E−04)	3.43E−03 (3.20E−04)	−8.50E−03 (1.30E−03)	−4.32E−03 (3.05E−04)
	${\alpha }_{3}{\beta }_{3}$	2.88E−03 (1.17E−04)	−1.15E−02 (4.25E−04)	2.38E−03 (1.15E−04)	6.43E−04 (1.17E−04)	−1.38E−02 (4.22E−04)	−2.27E−03 (1.18E−04)
	${\alpha }_{4}{\beta }_{4}$	1.06E−02 (5.68E−04)	6.82E−03 (1.20E−03)	9.09E−03 (5.32E−04)	2.99E−03 (4.38E−04)	−4.25E−03 (1.26E−03)	−7.83E−03 (4.99E−04)
	${\alpha }_{5}{\beta }_{5}$	1.57E−02 (8.59E−04)	6.22E−03 (1.70E−03)	1.26E−02 (7.55E−04)	6.31E−03 (6.40E−04)	−8.25E−03 (1.96E−03)	−9.78E−03 (7.13E−04)
	${\alpha }_{6}{\beta }_{6}$	−9.35E−05 (3.98E−06)	−9.84E−06 (2.76E−06)	−1.16E−04 (3.92E−06)	−1.13E−04 (3.75E−06)	−6.49E−05 (2.45E−06)	−9.40E−05 (3.77E−06)
	${\alpha }_{7}{\beta }_{7}$	4.63E−03 (6.31E−05)	1.03E−03 (1.79E−05)	3.94E−03 (5.85E−05)	4.57E−03 (5.64E−05)	1.13E−03 (1.85E−05)	2.41E−03 (3.99E−05)
	${\alpha }_{8}{\beta }_{8}$	2.92E−05 (1.56E−06)	−1.46E−05 (2.31E−07)	−2.62E−05 (1.51E−06)	9.12E−07 (1.19E−06)	−1.37E−05 (7.91E−08)	−2.86E−05 (1.11E−06)

We also present the estimated FDR and power of mediation effects testing in Tables 2 and 3, where the nominal level is 0.05. The results indicate that both HIMA2 and HIMA can achieve valid FDR control. Furthermore, HIMA2 is more powerful than HIMA in selecting significant mediators, though the differences become smaller when sample size increases. We also note that as the correlation among the mediators becomes larger, both methods suffer in terms of power.

**Table 2:** FDR at significance level 0.05

	$\rho =0$				$\rho =0.25$			
	$p=1000$		$p=5000$		$p=1000$		$p=5000$	
Method	$n=300$	$n=600$	$n=300$	$n=600$	$n=300$	$n=600$	$n=300$	$n=600$
HIMA2	0.0110	0.0030	0.0634	0.0214	0.0094	0.0053	0.0569	0.0202
HIMA	0.0225	0.0149	0.0316	0.0316	0.0244	0.0238	0.0320	0.0301
HDMA	0.2067	0.2553	0.2994	0.3739	0.1880	0.2299	0.2712	0.3678

	$\rho =0.50$				$\rho =0.75$			
	$p=1000$		$p=5000$		$p=1000$		$p=5000$	
Method	$n=300$	$n=600$	$n=300$	$n=600$	$n=300$	$n=600$	$n=300$	$n=600$
HIMA2	0.0099	0.0039	0.0351	0.0129	0.0055	0.0026	0.0097	0.0025
HIMA	0.0322	0.0253	0.0339	0.0281	0.0325	0.0232	0.0306	0.0327
HDMA	0.1482	0.1764	0.2533	0.3174	0.0990	0.1211	0.1740	0.1816

**Table 3:** Power at significance level 0.05

	$\rho =0$				$\rho =0.25$			
	$p=1000$		$p=5000$		$p=1000$		$p=5000$	
Method	$n=300$	$n=600$	$n=300$	$n=600$	$n=300$	$n=600$	$n=300$	$n=600$
HIMA2	0.8640	0.9608	0.8024	0.9392	0.8440	0.9512	0.8076	0.9364
HIMA	0.7760	0.9464	0.6192	0.8872	0.7800	0.9480	0.6472	0.9020
HDMA	0.8928	0.9848	0.7680	0.9496	0.9032	0.9880	0.8236	0.9652

	$\rho =0$.50				$\rho =0.75$			
	$p=1000$		$p=5000$		$p=1000$		$p=5000$	
Method	$n=300$	$n=600$	$n=300$	$n=600$	$n=300$	$n=600$	$n=300$	$n=600$
HIMA2	0.7996	0.9180	0.7596	0.9096	0.6612	0.8200	0.6416	0.8072
HIMA	0.7672	0.9252	0.6412	0.8876	0.5860	0.7584	0.5244	0.7232
HDMA	0.9052	0.9816	0.8136	0.9560	0.8436	0.9452	0.7900	0.9208

Per suggestion from a reviewer, we compare our method to HDMA [30], which was developed along the lines of HIMA but adopts the de-biased Lasso method in Step 2. However, no multiple testing adjustment was used in HDMA for inference. As a result, HDMA suffers from poor FDR control albeit with higher power as shown in Tables 2 and 3.

Per suggestion from a reviewer, similar to our real data analysis, we also consider a setting with 2 significant mediators, i.e.: ${\beta }_{1}=0.15,$ ${\beta }_{2}=0.3,$ ${\beta }_{3}=0.1,$ ${\beta }_{4}=0,$ and ${\beta }_{j}=0$ for all other $j$’s; ${\alpha }_{1}=0.15,$ ${\alpha }_{2}=0.3,$ ${\alpha }_{3}=0,$ ${\alpha }_{4}=0.1,$ and ${\alpha }_{j}=0$ for all other $j$’s. As shown in the supplementary materials (Additional file 1: Tables S1, S2 and S3), we observe similar results to those in Tables 1, 2 and 3. We note that the results from HIMA and HIMA2 are more close to each other when the correlation is high ($\rho =0.75)$.

Per suggestion from a reviewer, we use the standardized coefficient estimates in the SIS step, but the results are close to those without standardization (results available upon request).

Finally, we notice that in Tables 2 and Additional file 1: Table S2, the FDR of HIMA2 decreases with sample size. This also happens with HIMA, though to a less magnitude.

## Application

We apply our method to the Coronary Artery Risk Development in Young Adults (CARDIA) Study, an ongoing longitudinal cohort examining the development and determinants of clinical and subclinical cardiovascular disease and their risk factors [23]. A group of 5115 black and white men and women aged 18–30 years were enrolled in 1985–6 from 4 study centers: Birmingham, AL; Chicago, IL; Minneapolis, MN; and Oakland, CA. They were followed-up during 1987–1988 (Year 2), 1990–1991 (Year 5), 1992–1993 (Year 7), 1995–1996 (Year 10), 2000–2001 (Year 15), 2005–2006 (Year 20), 2010–2011 (Year 25), and 2015–2016 (Year 30).

We are interested in investigating how the DNA methylation (DNAm) markers mediate the relation between smoking and lung function. Due to budget limitation, 1200 individuals from the CARDIA participants at Year 15 were randomly selected for DNAm profiling using the Illumina MethylationEPIC Beadchip (*p* =  ~ 850,000 sites). The R package Enmix [31] was used to perform quality control, background correction, dye bias correction, quantile normalization (by probe types), and extreme outliers removal. Eventually, the DNAm measurements were obtained for a total of 1042 blood samples, which are treated as mediators in this study. The FEV1 (forced expiratory volume in 1 s) measured at Year 20 is considered as the lung function outcome. The number of cigarette packs/year in Year 10 is the exposure variable. We are interested in building the mediation pathway in sequence: smoking at Year 10 $\to$ High dimensional DNAm markers at Year 15 $\to$ lung function at Year 20.

Our analysis adjusts for age, height, weight, study center, gender, and race in Models (1) and (2). Additionally, we estimated the proportions of CD4 + T lymphocytes, CD8 + T lymphocytes, B lymphocytes, natural killer cells, monocytes, and granulocytes using [32], which are also adjusted in the models. To account for experimental batch effects and other technical biases, we derive surrogate variables from intensity data for non-negative internal control probes using principal components (PCs) analysis [31]. The top eight PCs, explaining 95.06% of the variation across the non-negative internal control probes, are also adjusted as covariates in the model. All the covariates are measured at Year 10.

After screening in Step 1, the average of the absolute values of correlation among CpGs is 0.25 (max 0.93). In Table 4, we present the summary results on selected mediators. For FDR < 0.05, HIMA2 identifies 2 CpGs: cg26331243 and cg19862839 as mediators. CpG cg26331243 is located in the body region of gene *CCDC33*, which is differentially expressed for tobacco smoke exposure [33, 34]. *CCDC33* is also linked to susceptibility to lung function disorders, e.g., pneumococcal meningitis [35] and SARS-CoV-2 infection [36]. Therefore, it is plausible that cg26331243 plays a role in regulating the expression of *CCDC33*, which in turn mediates the pathway from smoking to lung function.

**Table 4:** Summary of selected CpGs with mediation effects subject to FDR < 0.05

CpGs	Chromosome^1^	Position^a^	Proximal gene target^b^	${\widehat{\alpha }}_{k}(SE)$	${\widehat{\beta }}_{k}(SE)$	${\widehat{\alpha }}_{k}{\widehat{\beta }}_{k}$	FDR
cg26331243	chr15	74,550,946	CCDC33	−0.081 (0.016)	0.084 (0.027)	−0.0067	0.0345
cg19862839	chr17	59,543,726	TBX4	−0.082 (0.024)	0.059 (0.020)	−0.0049	0.0397

^a^Genome assembly GRCh37 (hg19)

^b^Based on UCSC RefGene

CpG cg19862839 is located in the body region of gene *TBX4*. Growing evidence has indicated that *TBX4* variants are associated with a wide spectrum of lung disorders [37, 38]. Patients with mutations in *TBX4* may also be more susceptible to cigarette smoking [39]. Therefore, we speculate that cg19862839 could participate in regulating the expression of *TBX4*, which also acts as a mediator between smoking and lung function.

In comparison, HIMA only identifies cg26331243 as a mediator with FDR < 0.05. Therefore, the proposed HIMA2 has better power to identify CpGs in high dimensional mediation analysis.

Finally, we note that cg05575921, which was identified in the normative aging study (NAS) [3], is not a significant mediator in the CARDIA study. In CARDIA, the estimate of $\alpha$ (from smoking to DNAm) is highly significant for cg05575921. However, the estimate of $\beta$ (from DNAm to FEV1) is not significant. This may be due to that participants in CARDIA were much younger (mean age 45 at Year 20, range 38–55) than NAS (mean age 74, range 55–100), when the lung function of CARDIA participants are more homogenous. Therefore, the association between DNAm to lung function at Year 20 may not be significant in CARDIA.

In the current analysis, there is a 5-year gap between the exposure and the mediator. A reviewer raised the concern on treatment-induced-mediator-outcome confounding. The life-course smoking trajectories for the majority of individuals were relatively stable before age 40–45, which corresponds to the Year 10–15 of our study cohort [40]. Although DNA methylation is modifiable by smoking, it is still a relatively stable biomarker over time [41]. Short-term exposure-induced covariates within a 5-year gap (if any) are unlikely to produce biologically functional changes in DNA methylation for us to detect as mediators.

## Conclusion and remarks

In this paper we proposed an improved method HIMA2 for high dimensional mediation analysis, which was shown to have better performance than HIMA [3] by numerical studies. We applied HIMA2 to the identification and testing of the DNA methylation mediating effects in the CARDIA study. Our method is relatively simple to implement, and can be widely used in high-dimensional mediation analyses.

Our method can be extended in several directions. First, we will consider how to address the correlation among DNA methylation markers to improve the inferential results, as shown in the Simulation Studies that both HIMA and HIMA2 lose power for high correlation. Second, it is of interest to incorporate the interaction terms between the exposure and the mediators in our model, i.e., the high dimensional moderated mediation analysis. Third, there has been an increasing interest and development in longitudinal studies of DNA methylation. We can also consider repeated measures of DNA methylation markers as mediators in our future research.

## Supplementary Information

**Additional file 1.** **Table S1:** Bias (MSE) for mediation effect estimates. **Table S2:** FDR at significance level 0.05. **Table S3:** Power at significance level 0.05.

## Data Availability

R package, source code, and simulation study are available at https://github.com/joyfulstones/HIMA2.
