# Supplementary material for: HIMA2: high-dimensional mediation analysis and its application in epigenome-wide DNA methylation data
Source: BMC Bioinformatics. 2022 Jul 25;23:296. doi: 10.1186/s12859-022-04748-1 (PMC9310002; doi:10.1186/s12859-022-04748-1)
Supplement: Supplementary file 1 — Additional file 1. Table S1: Bias (MSE) for mediation effect estimates. Table S2: FDR at significance level 0.05. Table S3: Power at significance level 0.05. [file 12859_2022_4748_MOESM1_ESM.docx]

Supplementary material for

**HIMA2: High-dimensional Mediation Analysis and its application in epigenome-wide DNA methylation data**

Chamila Perera^1^, Haixiang Zhang^2^, Yinan Zheng^3^, Lifang Hou^3^, Annie Qu^4^, Cheng Zheng^5^, Ke Xie^1^, Lei Liu^1*^

^1^Division of Biostatistics, Washington University in St. Louis, St. Louis, MO, 63110, USA

^2^Center for Applied Mathematics, Tianjin University, Tianjin 300072, China

^3^Department of Preventive Medicine, Northwestern University, Chicago, IL, 60611, USA

^4^Department of Statistics, University of California, Irvine, CA 92697, USA

^5^Department of Biostatistics, University of Nebraska Medical Center, Omaha, NE 68198, USA

*lei.liu@wustl.edu

Table S1: Bias (MSE) for mediation effect estimates

|  | | $\rho=0$ | | | | | |
| --- | --- | --- | --- | --- | --- | --- | --- |
|  |  | $p=1000$ | | | $p=5000$ | | |
|  |  | HIMA2 | HIMA | HDMA | HIMA2 | HIMA | HDMA |
| $n=300$ | $\alpha_{1}\beta_{1}$ | -7.12E-04 (9.28E-05) | -7.86E-03 (2.55E-04) | -7.38E-03 (1.53E-04) | -7.01E-03 (1.15E-04) | -1.56E-02 (3.59E-04) | -1.55E-02 (3.10E-04) |
|  | $\alpha_{2}\beta_{2}$ | -5.82E-03 (4.32E-04) | -1.38E-02 (6.87E-04) | -2.47E-02 (9.26E-04) | -2.54E-02 (9.25E-04) | -3.81E-02 (1.94E-03) | -4.46E-02 (2.19E-03) |
|  | $\alpha_{3}\beta_{3}$ | 2.03E-04 (8.45E-06) | -1.63E-05 (7.46E-06) | 3.10E-05 (5.44E-06) | -1.00E-04 (2.76E-06) | 6.95E-06 (1.48E-06) | -3.27E-05 (1.43E-06) |
|  | $\alpha_{4}\beta_{4}$ | 1.86E-03 (2.67E-05) | 1.30E-04 (3.62E-06) | 2.14E-04 (4.49E-06) | 8.60E-04 (1.86E-05) | -1.78E-05 (2.09E-06) | -3.24E-05 (1.13E-06) |
|  | $\alpha_{5}\beta_{5}$ | -3.10E-06 (8.95E-07) | 1.04E-05 (4.50E-07) | 3.61E-06 (5.06E-07) | 2.63E-05 (4.59E-07) | -1.01E-05 (6.15E-08) | 4.78E-06 (1.16E-07) |
| $n=600$ | $\alpha_{1}\beta_{1}$ | 5.38E-04 (5.55E-05) | -1.97E-04 (1.20E-04) | -4.22E-03 (6.23E-05) | -5.28E-03 (6.49E-05) | -8.65E-03 (1.73E-04) | -1.01E-02 (1.39E-04) |
|  | $\alpha_{2}\beta_{2}$ | -1.93E-03 (2.05E-04) | 2.50E-03 (3.13E-04) | -1.68E-02 (4.58E-04) | -2.09E-02 (6.12E-04) | -2.57E-02 (8.69E-04) | -3.82E-02 (1.58E-03) |
|  | $\alpha_{3}\beta_{3}$ | -2.48E-05 (4.24E-06) | -2.44E-05 (4.71E-06) | -4.92E-05 (3.09E-06) | -9.34E-05 (2.87E-06) | 2.42E-05 (2.54E-06) | -2.59E-06 (1.69E-06) |
|  | $\alpha_{4}\beta_{4}$ | 1.66E-03 (1.60E-05) | 2.68E-04 (4.44E-06) | 1.83E-04 (5.09E-06) | 5.65E-04 (9.31E-06) | 2.13E-05 (3.96E-07) | 2.75E-05 (9.40E-07) |
|  | $\alpha_{5}\beta_{5}$ | 2.40E-05 (4.07E-07) | -2.76E-06 (8.07E-08) | 7.44E-06 (1.57E-07) | 7.34E-06 (1.97E-07) | -1.22E-05 (7.23E-08) | -7.29E-06 (4.34E-08) |

|  | | $\rho=0.25$ | | | | | |
| --- | --- | --- | --- | --- | --- | --- | --- |
|  |  | $p=1000$ | | | $p=5000$ | | |
|  |  | HIMA2 | HIMA | HDMA | HIMA2 | HIMA | HDMA |
| $n=300$ | $\alpha_{1}\beta_{1}$ | -2.37E-04 (1.00E-04) | -7.61E-03 (2.63E-04) | -5.26E-03 (1.03E-04) | -5.20E-03 (1.06E-04) | -1.30E-02 (3.06E-04) | -1.06E-02 (1.67E-04) |
|  | $\alpha_{2}\beta_{2}$ | 3.69E-05 (4.56E-04) | -9.05E-03 (8.13E-04) | -2.05E-02 (8.16E-04) | -1.95E-02 (7.29E-04) | -3.21E-02 (1.53E-03) | -4.05E-02 (1.88E-03) |
|  | $\alpha_{3}\beta_{3}$ | 1.71E-04 (8.33E-06) | 7.08E-05 (6.06E-06) | 4.62E-05 (6.36E-06) | 1.50E-04 (5.25E-06) | 1.74E-04 (4.04E-06) | 1.64E-04 (3.47E-06) |
|  | $\alpha_{4}\beta_{4}$ | 3.33E-03 (3.88E-05) | 6.87E-04 (1.18E-05) | 1.20E-03 (1.22E-05) | 2.31E-03 (2.63E-05) | 3.13E-04 (4.58E-06) | 4.09E-04 (3.98E-06) |
|  | $\alpha_{5}\beta_{5}$ | 5.14E-05 (1.76E-06) | 3.03E-05 (7.93E-07) | -1.13E-05 (7.21E-07) | 1.54E-05 (3.26E-07) | 2.30E-06 (1.59E-09) | 5.72E-06 (3.37E-08) |
| $n=600$ | $\alpha_{1}\beta_{1}$ | 1.00E-04 (5.64E-05) | -2.26E-03 (1.60E-04) | -3.54E-03 (6.18E-05) | -4.98E-03 (6.70E-05) | -8.62E-03 (1.75E-04) | -9.01E-03 (1.10E-04) |
|  | $\alpha_{2}\beta_{2}$ | 2.74E-03 (2.36E-04) | 6.21E-03 (3.78E-04) | -1.09E-02 (3.39E-04) | -1.60E-02 (4.65E-04) | -2.18E-02 (7.30E-04) | -3.59E-02 (1.43E-03) |
|  | $\alpha_{3}\beta_{3}$ | -9.63E-05 (4.26E-06) | -1.20E-04 (4.69E-06) | -9.96E-05 (3.67E-06) | -5.22E-06 (2.77E-06) | 4.45E-05 (2.15E-06) | 1.64E-05 (1.65E-06) |
|  | $\alpha_{4}\beta_{4}$ | 2.59E-03 (2.10E-05) | 4.74E-04 (5.13E-06) | 1.39E-03 (8.70E-06) | 1.86E-03 (1.45E-05) | 2.91E-04 (3.24E-06) | 6.90E-04 (3.77E-06) |
|  | $\alpha_{5}\beta_{5}$ | 9.32E-06 (4.42E-07) | 5.98E-06 (6.42E-08) | -1.57E-07 (2.06E-07) | 8.39E-06 (1.55E-07) | -1.20E-07 (1.67E-08) | 1.62E-06 (4.43E-08) |

|  | | $\rho=0.5$ | | | | | |
| --- | --- | --- | --- | --- | --- | --- | --- |
|  |  | $p=1000$ | | | $p=5000$ | | |
|  |  | HIMA2 | HIMA | HDMA | HIMA2 | HIMA | HDMA |
| $n=300$ | $\alpha_{1}\beta_{1}$ | 6.88E-05 (1.27E-04) | -9.05E-03 (3.66E-04) | -3.16E-03 (1.21E-04) | -5.57E-03 (1.27E-04) | -1.50E-02 (3.68E-04) | -1.01E-02 (1.64E-04) |
|  | $\alpha_{2}\beta_{2}$ | 6.39E-03 (6.87E-04) | 3.32E-03 (1.39E-03) | -1.04E-02 (7.05E-04) | -1.07E-02 (7.21E-04) | -2.35E-02 (1.44E-03) | -3.42E-02 (1.59E-03) |
|  | $\alpha_{3}\beta_{3}$ | 2.69E-04 (6.59E-06) | 4.17E-05 (4.71E-06) | 2.45E-04 (5.65E-06) | 1.67E-04 (5.48E-06) | 4.99E-05 (4.95E-06) | 1.19E-04 (4.48E-06) |
|  | $\alpha_{4}\beta_{4}$ | 3.92E-03 (5.25E-05) | 1.10E-03 (2.03E-05) | 2.29E-03 (2.90E-05) | 3.41E-03 (3.77E-05) | 9.44E-04 (1.44E-05) | 1.91E-03 (1.70E-05) |
|  | $\alpha_{5}\beta_{5}$ | 4.52E-05 (1.95E-06) | 5.33E-05 (1.55E-06) | 3.55E-05 (1.30E-06) | -3.95E-05 (5.37E-07) | 1.14E-05 (2.34E-07) | 8.65E-06 (2.20E-07) |
| $n=600$ | $\alpha_{1}\beta_{1}$ | 6.19E-04 (6.47E-05) | -4.08E-03 (2.23E-04) | -1.51E-03 (5.90E-05) | -4.07E-03 (7.07E-05) | -9.96E-03 (2.35E-04) | -8.47E-03 (1.11E-04) |
|  | $\alpha_{2}\beta_{2}$ | 5.98E-03 (3.47E-04) | 1.39E-02 (9.32E-04) | -3.14E-03 (3.37E-04) | -1.10E-02 (3.82E-04) | -1.32E-02 (6.92E-04) | -2.99E-02 (1.08E-03) |
|  | $\alpha_{3}\beta_{3}$ | -6.98E-05 (3.57E-06) | -3.84E-05 (2.66E-06) | -9.75E-05 (3.55E-06) | 1.80E-04 (3.37E-06) | 1.22E-04 (2.81E-06) | 1.39E-04 (2.29E-06) |
|  | $\alpha_{4}\beta_{4}$ | 2.80E-03 (2.82E-05) | 3.41E-04 (3.99E-06) | 1.25E-03 (1.79E-05) | 1.81E-03 (2.07E-05) | 6.22E-04 (7.55E-06) | 1.10E-03 (1.11E-05) |
|  | $\alpha_{5}\beta_{5}$ | 5.24E-05 (5.75E-07) | 1.31E-05 (1.61E-07) | 1.45E-05 (4.22E-07) | 1.87E-05 (2.52E-07) | 2.27E-05 (1.31E-07) | 3.59E-05 (2.15E-07) |

|  | | $\rho=0.75$ | | | | | |
| --- | --- | --- | --- | --- | --- | --- | --- |
|  |  | $p=1000$ | | | $p=5000$ | | |
|  |  | HIMA2 | HIMA | HDMA | HIMA2 | HIMA | HDMA |
| $n=300$ | $\alpha_{1}\beta_{1}$ | -3.46E-03 (1.86E-04) | -1.76E-02 (4.99E-04) | -4.22E-03 (1.73E-04) | -3.98E-03 (1.85E-04) | -1.71E-02 (4.73E-04) | -7.39E-03 (1.82E-04) |
|  | $\alpha_{2}\beta_{2}$ | 2.42E-02 (1.59E-03) | 4.01E-02 (3.36E-03) | 1.22E-02 (1.24E-03) | 2.61E-03 (9.69E-04) | -6.15E-04 (1.44E-03) | -2.15E-02 (1.24E-03) |
|  | $\alpha_{3}\beta_{3}$ | 3.94E-05 (7.45E-06) | 3.84E-05 (4.10E-06) | 5.52E-05 (7.76E-06) | 6.65E-06 (5.61E-06) | -1.27E-05 (1.94E-06) | 1.34E-05 (4.71E-06) |
|  | $\alpha_{4}\beta_{4}$ | 4.35E-03 (8.15E-05) | 5.64E-04 (1.94E-05) | 3.46E-03 (7.26E-05) | 3.44E-03 (6.71E-05) | 6.88E-04 (1.72E-05) | 2.13E-03 (4.96E-05) |
|  | $\alpha_{5}\beta_{5}$ | 9.66E-05 (4.74E-06) | -7.18E-06 (1.50E-06) | -1.71E-05 (4.59E-06) | 1.33E-04 (2.75E-06) | 3.22E-05 (1.43E-06) | 1.61E-04 (2.65E-06) |
| $n=600$ | $\alpha_{1}\beta_{1}$ | -2.61E-03 (9.40E-05) | -1.43E-02 (4.33E-04) | -2.92E-03 (9.46E-05) | -3.53E-03 (1.04E-04) | -1.60E-02 (4.33E-04) | -6.11E-03 (1.13E-04) |
|  | $\alpha_{2}\beta_{2}$ | 1.91E-02 (8.74E-04) | 4.38E-02 (3.18E-03) | 1.46E-02 (7.33E-04) | 4.05E-03 (5.34E-04) | 2.03E-02 (1.31E-03) | -1.46E-02 (6.68E-04) |
|  | $\alpha_{3}\beta_{3}$ | -4.33E-05 (3.50E-06) | -1.01E-04 (2.26E-06) | -5.13E-05 (3.60E-06) | -2.87E-05 (3.59E-06) | 1.28E-05 (8.32E-07) | -5.32E-05 (3.06E-06) |
|  | $\alpha_{4}\beta_{4}$ | 4.29E-03 (5.86E-05) | 6.58E-04 (1.23E-05) | 3.67E-03 (5.46E-05) | 2.25E-03 (3.20E-05) | 4.83E-04 (7.85E-06) | 9.14E-04 (2.30E-05) |
|  | $\alpha_{5}\beta_{5}$ | -2.60E-05 (1.23E-06) | 1.44E-05 (5.12E-07) | -8.02E-06 (1.23E-06) | 7.92E-05 (1.08E-06) | 4.68E-05 (1.58E-07) | 5.81E-05 (8.94E-07) |

Table S2: FDR at significance level 0.05

|  | $\rho=0$ | | | | $\rho=0.25$ | | | |
| --- | --- | --- | --- | --- | --- | --- | --- | --- |
|  | $p=1000$ | | $p=5000$ | | $p=1000$ | | $p=5000$ | |
| Method | $n=300$ | $n=600$ | $n=300$ | $n=600$ | $n=300$ | $n=600$ | $n=300$ | $n=600$ |
| HIMA2 | 0.008 | 0.003 | 0.039 | 0.011 | 0.009 | 0.003 | 0.042 | 0.019 |
| HIMA | 0.021 | 0.022 | 0.026 | 0.019 | 0.029 | 0.016 | 0.035 | 0.022 |
| HDMA | 0.411 | 0.486 | 0.536 | 0.623 | 0.376 | 0.447 | 0.497 | 0.604 |

|  | $\rho=0.50$ | | | | $\rho=0.75$ | | | |
| --- | --- | --- | --- | --- | --- | --- | --- | --- |
|  | $p=1000$ | | $p=5000$ | | $p=1000$ | | $p=5000$ | |
| Method | $n=300$ | $n=600$ | $n=300$ | $n=600$ | $n=300$ | $n=600$ | $n=300$ | $n=600$ |
| HIMA2 | 0.007 | 0.002 | 0.035 | 0.008 | 0.003 | 0.003 | 0.006 | 0.002 |
| HIMA | 0.030 | 0.019 | 0.035 | 0.036 | 0.027 | 0.018 | 0.037 | 0.022 |
| HDMA | 0.329 | 0.342 | 0.489 | 0.574 | 0.216 | 0.256 | 0.401 | 0.421 |

Table S3: Power at significance level 0.05

|  | $\rho=0$ | | | | $\rho=0.25$ | | | |
| --- | --- | --- | --- | --- | --- | --- | --- | --- |
|  | $p=1000$ | | $p=5000$ | | $p=1000$ | | $p=5000$ | |
| Method | $n=300$ | $n=600$ | $n=300$ | $n=600$ | $n=300$ | $n=600$ | $n=300$ | $n=600$ |
| HIMA2 | 0.695 | 0.877 | 0.612 | 0.808 | 0.685 | 0.834 | 0.635 | 0.801 |
| HIMA | 0.628 | 0.872 | 0.488 | 0.772 | 0.620 | 0.850 | 0.511 | 0.764 |
| HDMA | 0.833 | 0.973 | 0.673 | 0.904 | 0.864 | 0.958 | 0.780 | 0.926 |

|  | $\rho=0$.50 | | | | $\rho=0.75$ | | | |
| --- | --- | --- | --- | --- | --- | --- | --- | --- |
|  | $p=1000$ | | $p=5000$ | | $p=1000$ | | $p=5000$ | |
| Method | $n=300$ | $n=600$ | $n=300$ | $n=600$ | $n=300$ | $n=600$ | $n=300$ | $n=600$ |
| HIMA2 | 0.616 | 0.784 | 0.586 | 0.750 | 0.497 | 0.589 | 0.462 | 0.598 |
| HIMA | 0.575 | 0.778 | 0.483 | 0.682 | 0.514 | 0.584 | 0.463 | 0.564 |
| HDMA | 0.820 | 0.954 | 0.725 | 0.904 | 0.703 | 0.837 | 0.647 | 0.832 |
